# Experimental Investigation of the Time Course Effects of Acute Exercise on False Episodic Memory

**DOI:** 10.3390/jcm7070157

**Published:** 2018-06-21

**Authors:** Ali Siddiqui, Paul D. Loprinzi

**Affiliations:** Exercise Psychology Laboratory, Physical Activity Epidemiology Laboratory, Department of Health, Exercise Science and Recreation Management, 229 Turner Center, School of Applied Sciences, The University of Mississippi, Oxford, MS 38677, USA; azsiddiq@go.olemiss.edu

**Keywords:** confabulation, exercise, memory, physical activity

## Abstract

Previous experimental work suggests that acute exercise may positively influence the accurate recall of past episodic events. However, few studies have examined whether acute exercise also reduces the number of false episodic memories. We evaluated this paradigm in conjunction with an examination of the temporal effects of acute exercise, which have previously been shown to play an important role in subserving episodic memory function. Twenty young adults participated in three experimental visits, including a non-exercise control visit, a visit involving an acute bout (20 min) of moderate-intensity exercise occurring prior to the memory task, and a visit involving an acute bout of exercise occurring during the encoding of the memory task. All visits were counterbalanced and occurred at least 24 h apart. The Deese–Roediger–McDermott (DRM) Paradigm, involving a separate word list trial for each visit, was employed to assess accurate and false episodic memory recall. For each visit, a short-term (immediate recall) and a long-term (25-min delay) memory recall was assessed. For both time points, the visit that involved exercise prior to encoding resulted in better short-term and long-term memory function (F(2) = 11.56, *p* < 0.001, η^2^_p_ = 0.38). For both time points, the control visit resulted in a greater number of false memories. These findings suggest that acute moderate-intensity exercise may help to increase the accurate recall of past episodic memories and may help to reduce the rate of false memories.

## 1. Introduction

Declarative memory includes the recall of fact-based information, referred to as semantic memory, in which episodic memory involves the retrospective recall of events or episodes occurring in a spatial or temporal pattern [1]. False episodic memory, or confabulated memories, refers to the perception of recalling a past event or episode, but the specific memory never occurred. Such experiences may occur as the result of the encoding or retrieval of a semantically related memory. Further, per the Fuzzy Trace Theory [2], when a memory is initially encoded, two memory traces are formed, namely, a verbatim trace and a gist trace. In comparison to the gist memory trace, the verbatim trace is more susceptible to biological decay. As a result, gist-like memories may be vulnerable to retrieval, ensuing a potential false memory. 

Of considerable interest to our group are the effects of exercise on episodic memory function. The field of exercise neurobiology has largely focused on working memory capacity and retrospective recall of actual events/episodes [1]. Previous work has detailed potential underlying mechanisms through which exercise may influence episodic memory [3]. Presently, few studies have examined the effects of acute exercise on false memory function [4]. The present experiment, which has been written as a brief report, evaluates this paradigm. Further, the potential temporal effects of acute exercise on false memory is examined, as previous work [5,6,7,8,9,10] in the declarative memory domain suggests that acute exercise, which occurs prior to memory encoding as opposed to during encoding or consolidation, may be optimal in enhancing memory function. 

## 2. Methods

### 2.1. Study Design

This study was approved by the Institutional Review Board at the authors’ University. Participants provided written consent before any data collection. A total of 20 participants completed three visits (around the same time of day), with these visits occurring at least 24 h apart. A counterbalanced, randomized, controlled, within-subject design was employed. The three counterbalanced visits included a control visit, walking prior to the memory task, and walking during the memory task. 

### 2.2. Participants

Similar to other experiments [11], participants included male and females from the ages of 18 to 35 yrs. Additionally, participants were excluded if they:

- Self-reported as a daily smoker [12,13]
- Self-reported being pregnant [14]
- Exercised within 5 h of testing [9]
- Consumed caffeine within 3 h of testing [15]
- Took medications known to influence cognition (e.g., antiepileptic meds, Adderall, herbal remedies) [16]
- Took medications used to regulate emotion (e.g., SSRI’s) [17]
- Had a concussion or head trauma within the past 30 days [18]
- Took marijuana or other illegal drugs within the past 30 days [19]
- Were considered a daily alcohol user (>30 drinks/month for women; >60 drinks/month for men) [20].

### 2.3. Recruitment

Participants were recruited by the student researcher using a non-probability convenience sampling approach at the authors’ university (i.e., student researcher proposed the study to students enrolled in university courses and sampled via word-of-mouth). 

### 2.4. Experimental Conditions

The two exercise conditions (walking before the memory task and walking during the memory task) included a 20-min bout of treadmill exercise (Woodway treadmill), followed by a 5-min recovery period. Participants were instructed to walk at a brisk walking pace, a speed as if they were late for catching the bus. A minimum speed of 3.0 mph (4.82 km/h) was set.

The control condition (time matched to the experimental conditions) involved playing a medium-level, online-administered Sudoku puzzle. Participants completed this puzzle prior to completing the memory task (described below). The website for this puzzle is located here: https://www.websudoku.com/.

### 2.5. Memory Assessments

The Deese–Roediger–McDermott (DRM) Paradigm [21] was used to assess false memory. For each visit, participants listened to a recording of a list of 15 words (separate word list for each visit); each word was read at a rate of one word per 1.5 s. After listening to the list, there was a 10-s pause, after which they re-listened to the list. The participants then recalled as many words from the list as they could remember.

The list was composed of associates (e.g., bed, rest, awake) of one non-presented word/lure (e.g., sleep). For example, if they indicated the word “sleep”, this was considered evidence of the construction of a false memory. Two outcome measures were derived from this memory assessment, including an episodic memory recall (number of correct words recalled; max = 15) and a false memory recall (whether they said the lure word; scored as 1 or 0, with 1 indicating they had a false memory). Both an episodic memory recall and false memory recall were evaluated immediately after the memory task, as well as 25 min later. Thus, a short- and long-term memory recall is reported herein.

### 2.6. Statistical Analysis

Analysis were computed using SPSS (v. 24; SPSS IBM, Armonk, NY, USA). To examine the time-course effects of exercise on memory, a 3 (conditions) × 2 (time points) repeated measures ANOVA was employed. Statistical significance was established as a nominal alpha of 0.05. Partial eta-squared (η^2^_p_) effect size estimates were calculated.

## 3. Results

Table 1 displays the characteristics of the sample. Participants were, on average, 21 years old; 40% were female; 75% were non-Hispanic white. The mean measured body mass index was 23.9 kg/m^2^. For the resting condition, heart rate remained in the mid- to upper-70s bpm during the control visit. For the visit involving exercise before the memory task, resting heart rate was 76 bpm and increased to 116 bpm during exercise. A similar exercise-induced heart rate response occurred for the “exercise during” condition (heart rate increased from 77 bpm to 116 bpm).

**Figure 1:**
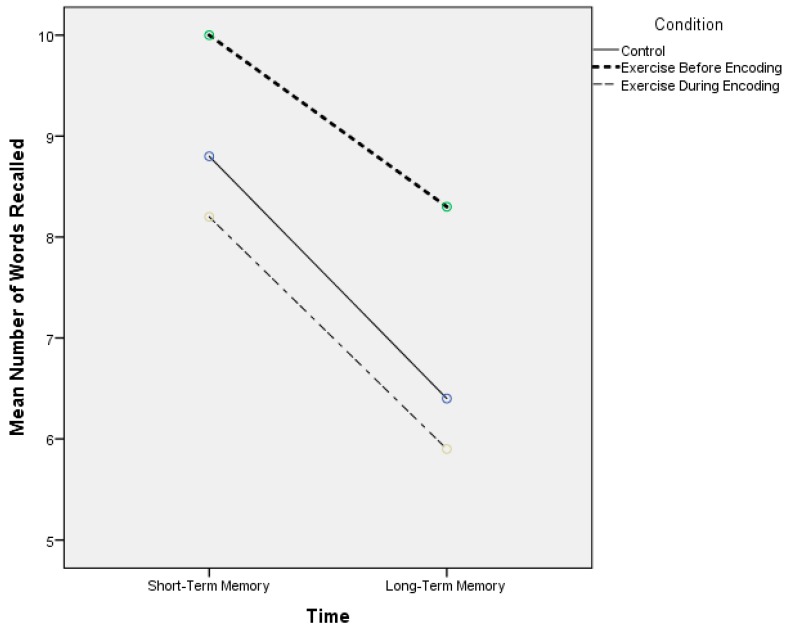
Episodic memory scores across the experimental conditions.

Table 2 displays the episodic memory scores across the three experimental conditions. For both short-term episodic memory recall and across the control, exercise before encoding, and exercise during encoding visits, respectively, the mean word recall was 8.80 (1.7), 10.0 (1.6), and 8.20 (1.6). For the delayed memory recall, the respective mean word recall scores were 6.40 (2.2), 8.30 (2.3), and 5.90 (1.7). Thus, for both time points, the visit that involved exercising prior to encoding resulted in better short-term and long-term memory function. These results are also graphically shown in Figure 1. There was a significant main effect for condition (F(2) = 11.56, *p* < 0.001, η^2^_p_ = 0.38), significant main effect for time (F(1) = 93.3, *p* < 0.001, η^2^_p_ = 0.83), but not significant condition × time interaction (F(2) = 1.52, *p* = 0.23, η^2^_p_ = 0.07).

**Figure 2:**
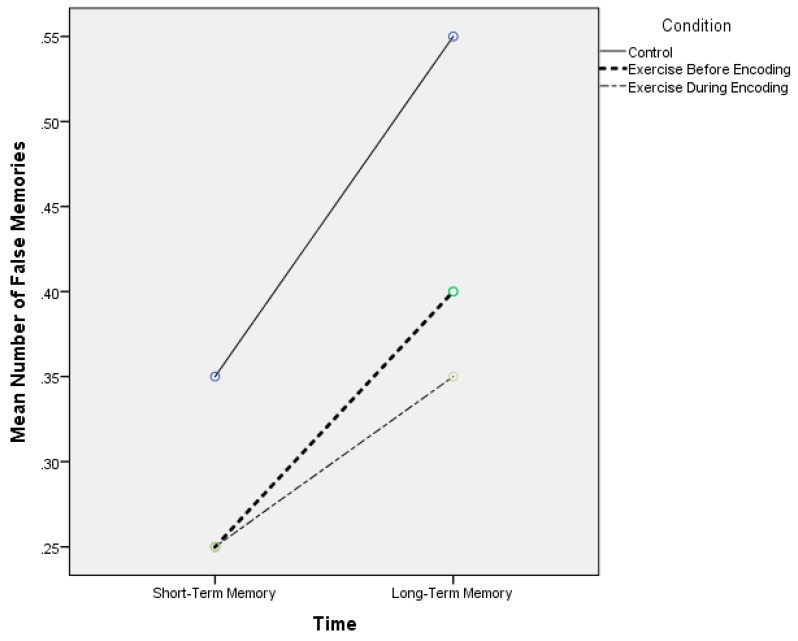
Mean number of false memories across the experimental conditions.

Table 2 also displays the false episodic memory scores across the three experimental conditions. For short-term false memory and across the control, exercise before encoding and exercise during encoding visits, respectively, the mean false memory score was 0.35 (0.48), 0.25 (0.44), and 0.25 (0.44). For the delayed false memory recall, the respective scores were 0.55 (0.51), 0.40 (0.50), and 0.35 (0.48). Thus, for both time points, the control visit resulted in a greater number of false memories. These results are graphically shown in Figure 2. There was no significant main effect for condition (F(2) = 1.11, *p* = 0.33, η^2^_p_ = 0.05), main effect for time (F(1) = 2.65, *p* = 0.11, η^2^_p_ = 0.12), or condition × time interaction (F(2) = 0.16, *p* = 0.85, η^2^_p_ = 0.01).

## 4. Discussion

This study evaluated the potential time course effects of acute moderate-intensity exercise on episodic memory function, including recall of true and false memories. In alignment with our previous work, along with others [9], with regard to the examination of both moderate-intensity [7] and high-intensity exercise [8], the present experiment demonstrates that acute exercise prior to the memory task may be optimal in enhancing episodic memory. That is, across the three conditions, the visit involving the exercise bout prior to the memory task resulted in the highest short- and long-term memory scores. In addition to true episodic memories, this investigation examined the time-course effects of acute exercise on false episodic memories. Although our results did not reach statistical significance, which aligns with our recent work that did not evaluate temporal effects of exercise [4], our findings provide suggestive evidence that acute exercise (either before or during encoding) was optimal to reduce false memories. That is, both exercise conditions (before or during encoding) had lower false memory scores when compared to the control condition.

Our findings also provide suggestive evidence that exercise temporality may have a differential effect on true and false memories, as the exercise before encoding condition had a higher number of true memories, but the two exercise conditions (exercising before and during encoding) had similar levels of false memories. Although these conclusions are speculative, exercising prior to memory encoding may help to prime the neurons for integration into the memory trace (engram), which would be optimal for true episodic memories, and exercising (at a moderate-intensity) both before and during memory encoding may have beneficial effects on executive function [22], which is important for attenuating false memories [23].

Our results suggest that acute exercise has a potential time-course effect in enhancing episodic memory and is supported on physiological grounds [3,24]. It is suggested that exercising prior to a memory task may help to optimize memory encoding by increasing psychological-based attentional resources, inducing neuronal excitability in the neurocircuitry of the memory system, and, in turn, the enhancement of long-term potentiation, a key postulated mechanism of episodic memory function. This temporal effect of exercise is supported by recent work which shows that exercise during memory encoding is associated with diminished hippocampus-dependent memory [25]. Further, the prefrontal cortex and the hippocampus are important brain structures in binding information together [26,27], which is critical for the prevention of false memory creation. Notably, acute exercise has been shown to influence the neural activity of these brain structures [3,28,29], providing some support for our findings that suggest a potential relationship between acute exercise and false memories.

Limitations of this study include the relatively small sample size, which may have influenced the statistical power of our study and possibly contributed to some of the non-statistically significant findings. Notably, particularly for the true episodic outcome measures, and as demonstrated by the effect size estimates, effects were fairly large. Nonetheless, future work on this topic should consider employing larger sample sizes. Strengths of this study include its novelty and experimental design.

In conclusion, this brief report supports emerging work suggesting a potential time-course effect of acute exercise on episodic memory function. Specifically, the findings of the present experiment demonstrate that acute exercise occurring prior to the memory task may be optimal in enhancing short- and long-term episodic memory function. Our experimental findings also provide some suggestive evidence that acute exercise may help to reduce the rate of false memories. Future experimental work on this topic is warranted.

## Figures and Tables

**Table 1:** Characteristics of the sample (N = 20).

Variable	Point Estimate	SD
Age, mean years	21.1	1.0
% Female	40	
Race-Ethnicity, %		
White	75.0	
Black	20.0	
Other	5.0	
BMI, mean kg/m^2^	23.9	3.4
Heart Rate, mean bpm		
Resting		
Control Visit	78.3	11.7
Exercise Before	75.4	12.5
Exercise During	78.0	11.4
Midway		
Control Visit	76.0	10.5
Exercise Before	113.2	17.7
Exercise During	115.9	15.8
Endpoint		
Control Visit	77.1	10.4
Exercise Before	116.8	17.1
Exercise During	115.6	17.0

**Table 2:** Memory scores across the three experimental conditions.

	Control	Exercise Before Memory Encoding	Exercise During Memory Encoding
**Immediate Memory Recall**			
# of words recalled (range = 0–15), mean	8.80 (1.7)	10.00 (1.6)	8.20 (1.6)
# of false memories (range = 0–1), mean	0.35 (0.48)	0.25 (0.44)	0.25 (0.44)
**Delayed Memory Recall**			
# of words recalled (range = 0–15), mean	6.40 (2.2)	8.30 (2.3)	5.90 (1.7)
# of false memories (range = 0–1), mean	0.55 (0.51)	0.40 (0.50)	0.35 (0.48)

## References

[1] Loprinzi P.D., Frith E., Edwards M.K., Sng E., Ashpole N. (2017). The Effects of Exercise on Memory Function Among Young to Middle-Aged Adults: Systematic Review and Recommendations for Future Research. Am. J. Health Promot..

[2] Reyna V.F., Brainerd C.J. (1998). Fuzzy-trace theory and false memory: New frontiers. J. Exp. Child Psychol..

[3] Loprinzi P.D., Edwards M.K., Frith E. (2017). Potential avenues for exercise to activate episodic memory-related pathways: A narrative review. Eur. J. Neurosci..

[4] Green D., Loprinzi P.D. (2018). Experimental Effects of Acute Exercise on Prospective Memory and False Memory. Psychol. Rep..

[5] Roig M., Thomas R., Mang C.S., Snow N.J., Ostadan F., Boyd L.A., Lundbye-Jensen J. (2016). Time-Dependent Effects of Cardiovascular Exercise on Memory. Exerc. Sport Sci. Rev..

[6] Roig M., Nordbrandt S., Geertsen S.S., Nielsen J.B. (2013). The effects of cardiovascular exercise on human memory: A review with meta-analysis. Neurosci. Biobehav. Rev..

[7] Sng E., Frith E., Loprinzi P.D. (2017). Temporal Effects of Acute Walking Exercise on Learning and Memory Function. Am. J. Health Promot..

[8] Frith E., Sng E., Loprinzi P.D. (2017). Randomized controlled trial evaluating the temporal effects of high-intensity exercise on learning, short-term and long-term memory, and prospective memory. Eur. J. Neurosci..

[9] Labban J.D., Etnier J.L. (2011). Effects of acute exercise on long-term memory. Res. Q. Exerc. Sport.

[10] Haynes J., Frith E., Sng E., Loprinzi P.D. (2018). The experimental effects of acute exercise on episodic memory function. Considerations for the timing of exercise. Psychol. Rep..

[11] Yanes D., Loprinzi P.D. (2018). Experimental Effects of Acute Exercise on Iconic Memory, Short-Term Episodic, and Long-Term Episodic Memory. J. Clin. Med..

[12] Jubelt L.E., Barr R.S., Goff D.C., Logvinenko T., Weiss A.P., Evins A.E. (2008). Effects of transdermal nicotine on episodic memory in non-smokers with and without schizophrenia. Psychopharmacology.

[13] Klaming R., Annese J., Veltman D.J., Comijs H.C. (2016). Episodic memory function is affected by lifestyle factors: A 14-year follow-up study in an elderly population. Neuropsychol. Dev. Cogn. B Aging Neuropsychol. Cogn..

[14] Henry J.D., Rendell P.G. (2007). A review of the impact of pregnancy on memory function. J. Clin. Exp. Neuropsychol..

[15] Sherman S.M., Buckley T.P., Baena E., Ryan L. (2016). Caffeine Enhances Memory Performance in Young Adults during Their Non-optimal Time of Day. Front. Psychol..

[16] Ilieva I.P., Hook C.J., Farah M.J. (2015). Prescription Stimulants’ Effects on Healthy Inhibitory Control, Working Memory, and Episodic Memory: A Meta-analysis. J. Cogn. Neurosci..

[17] Bauer E.P. (2015). Serotonin in fear conditioning processes. Behav. Brain Res..

[18] Wammes J.D., Good T.J., Fernandes M.A. (2017). Autobiographical and episodic memory deficits in mild traumatic brain injury. Brain Cogn..

[19] Hindocha C., Freeman T.P., Xia J.X., Shaban N.D.C., Curran H.V. (2017). Acute memory and psychotomimetic effects of cannabis and tobacco both ‘joint’ and individually: A placebo-controlled trial. Psychol. Med..

[20] Le Berre A.P., Fama R., Sullivan E.V. (2017). Executive Functions, Memory, and Social Cognitive Deficits and Recovery in Chronic Alcoholism: A Critical Review to Inform Future Research. Alcohol. Clin. Exp. Res..

[21] Roediger H.L., McDermott K.B. (1995). Creating false memories: Remebering words not presented in lists. J. Exp. Psychol. Learn. Mem. Cogn..

[22] Guiney H., Machado L. (2013). Benefits of regular aerobic exercise for executive functioning in healthy populations. Psychon. Bull. Rev..

[23] Devitt A.L., Schacter D.L. (2016). False memories with age: Neural and cognitive underpinnings. Neuropsychologia.

[24] Loprinzi P.D., Frith E. (2018). A brief primer on the mediational role of BDNF in the exercise-memory link. Clin. Physiol. Funct. Imaging.

[25] Soga K., Kamijo K., Masaki H. (2017). Aerobic Exercise During Encoding Impairs Hippocampus-Dependent Memory. J. Sport Exerc. Psychol..

[26] Nadel L., WIllner J. (1980). Context and conditioning: A place for space. Physiol. Psychol..

[27] Nadel L. (1991). The hippocampus and space revisited. Hippocampus.

[28] Tsujii T., Komatsu K., Sakatani K. (2013). Acute effects of physical exercise on prefrontal cortex activity in older adults: A functional near-infrared spectroscopy study. Adv. Exp. Med. Biol..

[29] Chang Y.K., Tsai C.L., Hung T.M., So E.C., Chen F.T., Etnier J.L. (2011). Effects of acute exercise on executive function: A study with a Tower of London Task. J. Sport Exerc. Psychol..

